# Relationship of being threatened or injured with a weapon in school with suicidal ideation and attempt among school students: a school-based study in Zhejiang Province, China

**DOI:** 10.1186/s12889-018-6302-z

**Published:** 2018-12-27

**Authors:** Hao Wang, Huaidong Du, Fiona Bragg, Jieming Zhong, Min Yu

**Affiliations:** 1 Department of NCDs Control and Prevention, Zhejiang Provincial Center for Diseases Control and Prevention, #3399 Binsheng road, Binjiang District, Hangzhou, 310051 Zhejiang Province China; 2 Clinical Trial Service Unit & Epidemiological Studies Unit (CTSU), Nuffield Department of Population Health, University of Oxford, Oxford, OX3 7LF UK; 3 Medical Research Council Population Health Research Unit, Nuffield Department of Population Health, University of Oxford, Oxford, OX3 7LF UK

**Keywords:** Suicidal ideation, Suicidal attempt, Threatened, Adolescents, Factors

## Abstract

**Background:**

Suicide is the second leading cause of death among 15–29 year old youths globally, and the third most common external cause of death in China. School bullying can cause serious consequences among adolescents, including psychosocial problems, low self-esteem and post-traumatic stress disorder. Most studies examining the associations between being bullied in school and suicide are from Western, developed countries. Moreover, few studies focus on being threatened or injured with a weapon, which may cause serious body injury. Our study aimed to explore the relationship of being threatened or injured with a weapon in school with suicidal ideation and attempt among middle and high school students in Zhejiang Province, China.

**Methods:**

A cross-sectional study of 23,543 students in grades 7–12 from 442 schools was carried out through an anonymous self-administered questionnaire between April and May 2017. Multivariable logistic regression models were used to examine the relationship of being threatened or injured with a weapon in school with suicidal ideation and attempt.

**Results:**

51.3% of participants were boys, and mean (SD) age was 15.6 (1.7) years. The overall prevalence of suicidal ideation and attempt were 16.1% (95%CI: 15.3–16.9) and 3.6% (95%CI: 3.3–4.0), respectively. Prevalence was higher among girls than boys (ideation: 19.1% vs. 13.3%. attempt: 4.4% vs. 2.9%). 13.2% of students reported being threatened or injured with a weapon in school in the past 12 months (95%CI: 11.9–14.5), higher among boys than girls (15.7% vs. 10.5%), and among rural students than urban students (13.9% vs. 11.6%). After adjustment for socio-demographic status, lifestyle factors, academic performance, self-reported health and mental health, the odds ratios for suicidal ideation and attempt among students who reported being threatened or injured with a weapon were 1.46 (1.31–1.61) and 1.68 (1.31–2.13) respectively, as compared with those who did not report being threatened or injured.

**Conclusions:**

Preventive measures for physical bullying in school need to be reinforced in China. Being threatened or injured with a weapon is associated with both suicidal ideation and attempt among middle and high school students. Suicide prevention should be an important component of psychological interventions for threatened or injured students.

## Background

Suicide is a serious public health problem worldwide and is responsible for more than 0.8 million deaths annually. In 2012, approximately 76% of deaths due to suicide occurred in low- and middle-income countries including China [1]. Suicide is the second leading cause of death among 15–29 year olds globally [1]. There were nearly 120.7 thousand deaths from suicide in China in 2012, accounting for 15% of global suicide deaths [1]. Suicide rates in China decreased significantly during the past 30 years [2, 3]. However, it remains the third most common external cause of death, following road traffic accidents and falls [4]. Between 2006 and 2016, mortality due to suicide among children and adolescents aged 10–14 years in urban areas increased rapidly, from 0.24 to 0.98 per 100,000, and in rural areas from 0.97 to 0.85 per 100,000. Mortality due to suicide among adolescents aged 15–19 years decreased from 1.80 to 1.56 per 100,000 in urban areas, and from 3.47 to 2.52 per 100,000 in rural areas [5]. In Chinese traditional culture, one should not deliberately hurt oneself. As a Chinese saying states, the body, hair and skin have all been received from the parents, and so one doesn^’^t dare damage them; this is the beginning of filial piety. While in Western countries, mental health disorders account for more than 90% of suicides, only about half of suicides were related to mental health disorders in China [6]. Easy access to pesticides increases the risk of suicide in rural areas of China [7], while lack of medical care and facilities increases the possibility of completed suicide [8]. Hence, it is necessary to study the epidemiological profile of suicide behaviours among Chinese students.

Suicidal ideation and suicide attempt are recognized as important predictors and indicators of subsequent completed suicide [9, 10]. A meta-analysis including 320,375 students included in 40 studies conducted in China found that the prevalence of suicidal ideation among high school students was 18.0% (95%CI: 16.6–19.5) [11]. The epidemiology of suicidal ideation and attempt involves a large number of risk factors, including socio-demographic factors (e.g., gender, family income and family structure) [12, 13], behavioural factors (cigarette use, alcohol use, illicit drug use, inadequate physical activity and ever having sexual intercourse) [14–16], mental health (e.g., sadness, loneliness and depression) [15, 17, 18], poor physical health [19, 20] and poor academic performance [21].

School bullying refers to any unwanted aggressive behaviours towards one youth by another youth or group of youths, who are not siblings or current dating partners, involving an observed or perceived power imbalance. These behaviours are repeated, or have the potential to be repeated, over time [22]. Bullying may comprise physical or non-physical bullying. The latter includes verbal bullying (e.g., verbal insulting, slandering, gossiping and taunting etc.) and non-verbal bullying (e.g., obscene gesture, hiding belongings, deliberate excluding from a group or activity, and social marginalization etc.) [23]. School bullying may have serious consequences among adolescents, including psychosocial problems (e.g., depression, loneliness and anxiety) [24, 25], low self-esteem [26] and post-traumatic stress disorder [27].

Physical bullying among adolescents is less normative compared to other types of bullying and therefore associated with more severe outcomes [28]. Being threatened or injured with a weapon is one of the most serious forms of physical bullying [29]. The prevalence of being threatened or injured with a weapon on school property among high school students ranged from 4.8% (Massachusetts) to 12.8% (Louisiana) in the USA in 2017 [30], but little is known about the epidemiological profile of being threatened or injured with a weapon among students in China.

More recently, a large number of articles have focused on the effect of being bullied in school on adolescent suicide [31]. However, most studies are from Western, developed countries [32–35]. Moreover, the association of being bullied in school with risk of suicidality is still controversial. Some studies have observed positive associations [34, 35], while others have found null associations [36, 37]., A latest longitudinal study indicated that the association between victimization and suicide behaviours relates to the specific type of victimization. Relational, but not verbal or physical, victimization significantly increased the likelihood of experiencing suicidal ideation. Conversely, physical victimization, but not verbal or relational, significantly increased the likelihood of suicide attempt [38]. A study of 20,509 high school students from China found that being bullied was associated with an increased risk of suicidal ideation. However, this study did not explore the relationship between being bullied and suicidal attempt [39]. Furthermore, few studies have examined the associations of being threatened or injured with a weapon, which will additionally cause direct body injury. The current study was designed with the aim of examining the relationship of being threatened or injured with a weapon in school and suicidal ideation with attempt among students in Zhejiang Province.

## Participants and methods

### Survey design and participants

Details of the study design, sampling, and procedure have been described previously [40]. In brief, a three-stage sampling design was utilized. In stage one, 30 counties were sampled randomly from all 90 counties. In stage two, 10 classes of middle school, 5 classes of academic high school, and 5 classes of vocational high school were selected randomly within each chosen county. In stage three, all students in the chosen classes were invited to participate in the study. The survey questionnaire was modelled on existing surveys including the Youth Risk Behaviour Survey (YRBS), conducted by the Centers for Disease Control and Prevention (CDC) [41], and the international Global School-based Student Health Survey, supported by the World Health Organization [42]. Survey questions addressed demographic characteristics (including age, gender, parental educational level, and parental marital status), tobacco and alcohol use, physical activity, violence, suicidal behaviours, and sexual behaviours. Strict quality control measures were undertaken to ensure data quality. Participants were asked to complete a questionnaire in the classroom in the absence of school teachers, with a distance of at least one meter between students’ seat. All participants were advised, by trained interviewers, that there were no so-called “correct” or “incorrect” answers, that the questions should be answered honestly and accurately, and that their answers were unrelated to their academic performance and would be kept confidential (including from their school teachers and parents). The questionnaire was put into sealed box by participants after completion.

### Measures

#### Outcome variables

Suicidal ideation was assessed by a yes/no response to the question “During the past 12 months, did you ever seriously consider attempting suicide?” Suicidal attempt was assessed through the question: “During the past 12 months, how many times did you actually attempt suicide?” Response options included: “None”, “1 time”, “2–3 times”, “4–5 times” and “6 or more times.” Suicidal attempt was defined as at least once in the past 12 months.

#### Exposure variables

Being threatened or injured with a weapon in school was assessed with the question: “During the past 12 months, how many times has someone threatened or injured you with a weapon such as a club or knife on school property?” Answer options included: “None”, “1 time”, “2-3times”, “4–5 times”, “6–7 times”, “8–9 times”, “10–11 times” and “12 or more times”. Students who reported one or more time were classified as being threatened or injured with a weapon.

#### Covariates

Covariate question and response options (including parental education, parental marital status, cigarette use, alcohol use, physical activity, academic performance, self-reported health, sexual experience, loneliness) have been reported previously [40, 43]. Sadness was assessed through the question: “During the past 12 months, did you ever feel so sad or hopeless almost every day for two weeks or more in a row that you stopped doing some usual activities?” Answer options were “yes” and “no”.

### Statistical analysis

Statistical analyses were performed using SAS software V.9.3. A weighting factor was applied at the student-level to adjust for non-response and for the varying probabilities of selection. The weight used for estimation in this survey is given by: W = W1 × W2 × f1 × f2. W1 = the inverse of the probability of selecting the county. W2 = the inverse of the probability of selecting the classroom within the county. f1 = a student-level nonresponse adjustment factor calculated by class. f2 = a post-stratification adjustment factor calculated by grade [44]. Continuous variables were summarised as mean ± standard deviation. The prevalence of suicidal ideation and attempt, and being threatened or injured with a weapon in school, were summarised as percent and 95% confidence intervals (CI). Weighted prevalence was calculated using the PROC SURVEYFREQ procedure and its difference between groups was compared using Rao-Scott Chi-square test. To evaluate the associations between being threatened or injured with a weapon in school and suicidal ideation and attempt, multivariable logistic regression analyses were performed using the PROC SURVEYLOGISTC procedure, to take into account the complex survey sampling methods. Potential confounding factors, including socio-demographic status, lifestyle factors, academic performance, self-reported health and mental health were adjusted in different models. Odds ratios were calculated using four multivariable logistic regression models. In model 1, odds ratios were adjusted for age (≤13 years, 14–15 years and ≥ 16 years), sex (boys and girls), region (urban and rural), and type of school (middle school, academic high school and vocational high school). Model 2 included additional adjustment for parental and maternal education level (middle school or below, high school, college or above and unknown) and parental marital status (married and others). Model 3 included additional adjustment for cigarette smoking (current smoker and non-smoker), alcohol drinking (current drinker and non-drinker), physical activity (none, 1–2 days/week, 3–5 days/week and 6–7 days/week), academic performance (excellent, middle and bad), self-reported health status (very good/good, fair, very bad/bad and unknown) and sexual experience (yes and no). Model 4, additionally adjusted for loneliness (never/occasional, sometimes and often/always) and sadness (yes and no). All statistical tests were two tailed, and *P* values < 0.05 were considered statistically significant.

## Results

### Descriptive statistics

A total of 24,157 students from 442 schools were invited to participate. Due to missing or incomplete questionnaires or refusal to participate, 23,543 eligible students (response rate 97.5%) were included in the final analyses. 12,068 (51.3%) were boys, and mean age was 15.6 years. 12,207 (51.9%) students were from middle schools, 6477 (27.5%) were from academic high schools, and 4859 (20.6%) came from vocational high schools.

Compared with those who reported not being threatened or injured, students who reported being threatened or injured with a weapon in school were more likely to be boys (61.8% vs. 50.3%) (Table 1), to come from a rural area (71.9% vs. 67.6%), to have poor academic performance (31.1% vs. 26.4%), to smoke cigarettes (8.6% vs. 5.0%), to drink alcohol (31.1% vs. 21.5%), to describe their health as very bad or bad (9.0% vs. 5.2%), to report previous sexual experiences (6.4% vs. 3.5%), to feel lonely (18.3% vs. 10.2%) or sad (25.8% vs. 13.9%), and were less likely to come from intact families (88.0% vs. 90.4%). No significant difference was observed between parental education level and being threatened or injured with a weapon in school (*P* > 0.05).

**Table 1:** Participant characteristics by history of being threatened or injured with a weapon (*N* = 23,543)

Characteristics	Total	Without being threatened or injured	Being threatened or injured	x^2a^	*P* value
	(*N* = 23,543)	(*N* = 20,502)	(*N* = 3041)		
Age range (years)				60.9	<.0001
≤ 13	5159 (20.9)	4208 (19.5)	951 (30.5)		
14–15	8030 (34.3)	6878 (33.8)	1152 (37.6)		
≥ 16	10,354 (44.8)	9416 (46.7)	938 (31.9)		
Sex				83.0	<.0001
Boys	12,068 (51.8)	10,196 (50.3)	1872 (61.8)		
Girls	11,475 (48.2)	10,306 (49.7)	1169 (38.2)		
Area				3.9	0.04
Urban	9022 (31.8)	7990 (32.4)	1032 (28.1)		
Rural	14,521 (68.2)	12,512 (67.6)	2009 (71.9)		
Types of school				76.5	<.0001
Middle school	12,207 (51.8)	10,210 (49.7)	1997 (65.4)		
Academic high school	6477 (26.1)	5945 (27.6)	532 (16.3)		
Vocational high school	4859 (22.1)	4347 (22.7)	512 (18.3)		
Parental marital status				10.7	0.0011
Married	21,151 (90.1)	18,482 (90.4)	2669 (88.0)		
Others	2392 (9.9)	2020 (9.6)	372 (12.0)		
Paternal education level				1.9	0.59
Middle or below	13,568 (60.0)	11,762 (59.8)	1806 (61.4)		
High school	5100 (20.8)	4457 (20.9)	643 (19.6)		
College or above	3129 (11.4)	2756 (11.5)	373 (11.1)		
Unknown	1746 (7.8)	1527 (7.8)	219 (7.9)		
Maternal education level				0.20	0.98
Middle or below	14,530 (63.9)	12,627 (64.0)	1903 (63.5)		
High school	4363 (17.7)	3802 (17.6)	561 (18.1)		
College or above	2736 (10.0)	2403 (10.0)	333 (10.0)		
Unknown	1914 (8.4)	1670 (8.4)	244 (8.4)		
Physical activity (d/wk)				10.5	0.01
0	4883 (21.5)	4306 (21.8)	577 (19.7)		
1–2	5690 (24.8)	4995 (25.1)	695 (23.1)		
3–5	8050 (33.0)	6979 (32.9)	1071 (34.1)		
6–7	4920 (20.7)	4222 (20.2)	698 (23.1)		
Academic performance				18.6	<.0001
Excellent	5448 (22.5)	4782 (22.8)	666 (20.7)		
Middle	11,765 (50.5)	10,331 (50.8)	1434 (48.2)		
Bad	6330 (27.0)	5389 (26.4)	941 (31.1)		
Current smoking	1247 (5.5)	984 (5.0)	263 (8.6)	43.8	<.0001
Current drinking	5345 (22.8)	4382 (21.5)	963 (31.1)	49.1	<.0001
Self-reported health				90.0	<.0001
Very good/good	12,415 (51.8)	11,002 (52.7)	1413 (45.8)		
Fair	9563 (41.4)	8256 (41.1)	1307 (43.4)		
Very bad/bad	1293 (5.7)	1029 (5.2)	264 (9.0)		
Unknown	272 (1.1)	215 (1.0)	57 (1.8)		
Sexual experience	861 (3.9)	669 (3.5)	192 (6.4)	35.1	<.0001
Loneliness				220.7	<.0001
Never/occasional	15,122 (64.1)	13,574 (66.2)	1548 (49.9)		
Sometimes	5783 (24.7)	4844 (23.6)	939 (31.8)		
Often/always	2638 (11.2)	2084 (10.2)	554 (18.3)		
Sadness	3676(15.4)	2893 (13.9)	783 (25.8)	142.5	<.0001

Number in brackets are weighted proportions. ^a^ Rao-Scott Chi-Square

### Prevalence of suicidal ideation and attempt

The overall prevalence of suicidal ideation was 16.1% (95%CI: 15.3–16.9) (Table 2), higher among girls than boys (19.1% vs. 13.3%) (*P* < 0.0001). There was no statistically significant difference in the prevalence of suicidal ideation between urban and rural areas (16.5% vs. 15.9%) (*P* = 0.43). The prevalence among students aged ≤13 years, 14–15 years and ≥ 16 years was 15.9% (95%CI: 14.4–17.4), 17.9% (95%CI: 16.7–19.1) and 14.8% (95%CI: 13.7–15.9), respectively. The corresponding prevalence among students attending middle school, academic high school and vocational high school was 16.9% (95%CI: 15.8–18.0), 16.6% (95%CI: 15.3–17.9) and 13.7% (95%CI: 11.9–15.5), respectively (*P* = 0.0048).

**Table 2:** Weighted prevalence of suicidal ideation and attempt by different subgroups

Characteristics	Suicidal ideation			Suicidal attempt		
	Prevalence (95%CI)^a^	x^2b^	*P* value	Prevalence (95%CI)^a^	x^2b^	*P* value
Age range (years)		16.8	0.0002		29.4	<.0001
≤ 13	15.9 (14.4–17.4)			4.1 (3.5–4.8)		
14–15	17.9 (16.7–19.1)			4.4 (3.9–4.9)		
≥ 16	14.8 (13.7–15.9)			2.8 (2.3–3.2)		
Sex		88.2	<.0001		45.5	<.0001
Boys	13.3 (12.3–14.2)			2.9 (2.5–3.2)		
Girls	19.1 (18.1–20.2)			4.4 (3.9–4.9)		
Area		0.6	0.43		0.45	0.50
Urban	16.5 (15.3–17.8)			3.5 (3.0–3.9)		
Rural	15.9 (14.9–16.9)			3.7 (3.2–4.2)		
Types of school		10.7	0.0048		27.2	<.0001
Middle school	16.9 (15.8–18.0)			4.4 (3.9–4.9)		
Academic high school	16.6 (15.3–17.9)			2.4 (1.9–2.8)		
Vocational high school	13.7 (11.9–15.5)			3.3 (2.6–4.1)		

^a^Based on the weighted data. ^b^: Rao-Scott Chi-Square, *CI* Confidence Interval

The overall prevalence of suicide attempt was 3.6% (95%CI: 3.3–4.0) (Table 2), higher among girls than boys (4.4% vs. 2.9%) (*P* < .0001). No regional difference in suicidal attempt was found (urban: 3.5% vs. rural: 3.7%) (*P* = 0.50). The prevalence of suicidal attempt among students aged ≤13 years, 14–15 years and ≥ 16 years was 4.1% (95%CI: 3.5–4.8), 4.4% (95%CI: 3.9–4.9) and 2.8% (95%CI: 2.3–3.2), respectively. The corresponding figures for students attending middle school, academic high school and vocational high school were 4.4% (95%CI: 3.9–4.9), 2.4% (95%CI: 1.9–2.8) and 3.3% (95%CI: 2.6–4.1), respectively (*P* < .0001).

### Prevalence of being threatened or injured with a weapon in school

13.2% (95%CI: 11.9–14.5) of students reported being threatened or injured with a weapon in school in the past 12 months (Table 3), higher among boys (15.7% [95%CI: 14.2–17.2]) than girls (10.5% [95%CI: 9.1–11.8]), and among rural than urban students (13.9% vs. 11.6%). The prevalence of being threatened or injured with a weapon in school among students attending middle school, academic high school and vocational high school was 16.7% (95%CI: 14.6–18.7), 8.2% (95%CI: 7.0–9.4) and 10.9 (95%CI: 9.2–12.6), respectively (*P* < .0001). The prevalence of being threatened or injured with a weapon increased with the decreasing age (*P* < .0001). There was no regional difference in the prevalence of being threatened or injured with a weapon in school among boys (urban: 14.5% vs. rural: 16.3%) (*P* = 0.18). However, rural girls had a higher prevalence than their urban counterparts (11.3% vs. 8.6%) (*P* = 0.02).

**Table 3:** Weighted prevalence of being threatened or injured with a weapon in school by different subgroups

Characteristics	Overall			Boys			Girls		
	Prevalence (95%CI)^a^	x^2^	*P* value	Prevalence (95%CI)^a^	x^2^	*P* value	Prevalence (95%CI)^a^	x^2^	*P* value
Age range (years)		289.5^c^	<.0001		154.3^c^	<.0001		135.0^c^	<.0001
≤ 13	19.2 (15.9–22.4)			22.5 (18.8–26.2)			15.5 (12.2–18.8)		
14–15	14.5 (12.4–16.5)			17.0 (14.9–19.1)			11.7 (9.5–13.9)		
≥ 16	9.4 (8.4–10.4)			11.5 (10.2–12.8)			7.1 (5.8–8.5)		
Area		3.95^b^	0.04		1.76^b^	0.18		5.5^b^	0.02
Urban	11.6 (10.3–12.9)			14.5 (12.9–16.1)			8.6 (7.1–10.0)		
Rural	13.9 (12.1–15.8)			16.3 (14.2–18.3)			11.3 (9.5–13.2)		
Types of school		76.5^b^	<.0001		64.0^b^	<.0001		32.6^b^	<.0001
Middle school	16.7 (14.6–18.7)			19.6 (17.3–21.8)			13.4 (11.3–15.6)		
Academic high school	8.2 (7.0–9.4)			10.3 (8.7–11.9)			6.2 (4.9–7.5)		
Vocational high school	10.9 (9.2–12.6)			12.8 (10.9–14.8)			8.7 (6.2–11.3)		

^a^Based on the weighted data. ^b^: Rao-Scott Chi-Square, ^c^:Trend for Chi-Square, *CI* Confidence Interval

### Logistic regression analysis

After adjusting for age, sex, region, and type of school (Table 4), compared to those who reported not being threatened or injured, the odds ratios for suicidal ideation and attempt were 2.29 (2.08–2.63) and 2.93 (2.34–3.67) (*P* < 0.0001 for all), respectively, among students who reported being threatened or injured with a weapon in school. After further adjustment for parental education level and marital status, lifestyle factors, academic performance, and health status, the corresponding odds ratios were attenuated to 1.89 (1.72–2.09) and 2.16 (1.72–2.72), respectively (*P* < 0.0001 for all). After additional adjustment for mental health (i.e., loneliness and sadness). These associations were further markedly attenuated, with corresponding odds ratios of 1.46 (1.31–1.61) and 1.68 (1.31–2.13), respectively (*P* < 0.0001 for all). No sex-difference was observed in the associations of being threatened or injured with a weapon and suicidal ideation (*P* = 0.61) or attempt (*P* = 0.07).

**Table 4:** Adjusted odds ratios of suicidal behaviours associated with being threatened or injured with a weapon among school students in China

	Suicidal ideation				Suicidal attempt			
	Total	Boys	Girls	P^a^	Total	Boys	Girls	P^a^
Model 1	2.29 (2.08–2.63)	2.26 (1.96–2.62)	2.34 (2.04–2.68)	0.53	2.93 (2.34–3.67)	2.56 (1.89–3.46)	3.29 (2.49–4.35)	0.11
Model 2	2.27 (2.06–2.51)	2.24 (1.94–2.59)	2.31 (2.01–2.66)	0.54	2.89 (2.30–3.63)	2.55 (1.88–3.45)	3.23 (2.43–4.29)	0.11
Model 3	1.89 (1.72–2.09)	1.89 (1.63–2.17)	1.91 (1.65–2.21)	0.56	2.16 (1.72–2.72)	1.89 (1.37–2.62)	2.45 (1.87–3.22)	0.07
Model 4	1.46 (1.31–1.61)	1.46 (1.25–1.70)	1.47 (1.26–1.71)	0.61	1.68 (1.31–2.13)	1.43 (1.02–2.00)	1.93 (1.44–2.58)	0.07

Model 1, odds ratios were adjusted for age, sex, region, type of school. Model 2, additionally adjusted for parental and maternal education level and parental marital status. Model 3, additionally adjusted for cigarette smoking, alcohol drinking, physical activity academic performance, self-reported health status, sexual experience. Model 4, additionally adjusted for loneliness and sadness. ^a^P for gender heterogeneity

## Discussion

In this provincially representative study of middle and high school students in Zhejiang, China, we examined the prevalence of being threatened or injured with a weapon in school and suicidal ideation and attempt. We identified and quantified the relationship of being threatened or injured with a weapon in school and both suicidal ideation and attempt.

### Prevalence of suicidal ideation and attempt

Suicidal ideation involves a hierarchy of feelings from the thoughts of “Life is not worth living” to more serious articulation of a thought out plan. Suicidal attempt is defined as self-inflicted, potentially injurious behaviours with a nonfatal outcome, for which there was evidence of intent to die [45]. In the present study, the overall prevalence of suicidal ideation and attempt were 16.1 and 3.6% respectively, lower than the results of the 2015 USA-based YRBS (17.7 and 8.6%, respectively) [46]. Consistent with results from studies in Western countries [35, 46], our study indicated that the prevalence of suicidal ideation and attempt were higher among girls than among boys. Interestingly, national mortality surveillance in China demonstrated that men had a higher suicide rate than women in the general population [3]. This discrepancy may be explained by women adopting non-lethal means to attempt suicide [47, 48].

### Prevalence of being threatened or injured with a weapon in school

In the present study, 13.2% of students reported being threatened or injured with a weapon in school during the past 12 months, more than twice the reported prevalence in the USA (6.0%) [30]. This suggests that being threatened or injured with a weapon in school is common in China. This highlights the need for both the public and government to focus on the prevention of school bullying. Consistent with the previous US study [30], our study found that the prevalence of being threatened or injured with a weapon in school was higher among boys than girls. The possible explanation was that boys are more likely to be physically bullied [49]. While girls are more likely to be exposed to more covert forms of bullying (e.g., gossiping and social exclusion) [50]. Our study demonstrated that rural students were more likely to be threatened or injured with a weapon than urban students. There are several possible explanations for this phenomenon. First, there were more left-behind children in rural areas. In the absence of family care, children may be inclined to be violent [51]. Second, schools in rural areas may focus less on prevention and management of school bullying in comparison with schools in urban areas.

### Association of being threatened or injured with a weapon and suicide ideation and attempt

A 2013 meta-analysis of 41 studies found that being bullied was positively associated with suicidal ideation (OR = 2.34, 95%CI: 2.03–2.69) and suicide attempt among adolescents (OR = 2.94, 95%CI: 2.36–3.67) [31]. However, this study failed to ascertain the associations between different types of bullying victimization and suicidal behaviours. A longitudinal study of 2464 Norwegian adolescents aged 12–15 years observed that exposure to bullying during adolescence increased the risk of both suicidal ideation and attempt among boys and girls, and this effect persisted into young adulthood [52]. Another cohort study of 5302 Finnish children showed that the association between being bullied at age 8 years and later suicidal attempts varied by sex. After controlling for conduct and depression, the association remained significant among girls, but not among boys [53]. Our study found that being threatened or injured with a weapon in school was positively associated with risk of suicidal attempt among both girls and boys. The mechanisms underlying the associations with suicidal ideation and attempt remain unclear. One explanation may be that being threatened or injured represents a serious physical victimization and could cause depression, which, in turn, has been closely associated with suicidal ideation and attempt [54, 55]. Previous studies found that students who were once victims of bullying were more likely to experience depression [56, 57], and Brunstein Klomek et al. found a bidirectional association between victimization and depression [38].

There are a number of important implications of the findings of our study for prevention of adolescent suicide. First, although the Chinese government introduced a strategy for strengthening the comprehensive management of bullying, and launched an anti-bullying campaign in primary and middle schools in 2017, prevention of school bullying needs to be further reinforced in China. Students should be taught how to deal with school bullying, including being threatened or injured by others with a weapon. Second, students who report being threatened or injured with a weapon should be identified as being at-risk, and suicide prevention should be an important component of psychological support and interventions provided to these students.

### Strengths and limitations

The strengths of the current study include the provincially representative sample, a high response rate and a standardized procedure. The study questionnaire collates data on a range of socio-demographic, behavioral and mental health factors, which allowed us to better control confounding. Our study, however, had several limitations. First, the cross-sectional design limits establishment of the temporal relationship of being threatened or injured with a weapon in school with suicidal ideation and attempt, and it is not possible to establish the causality of the relationship. Second, all data were self-reported; some information might not be accurately reported due to recall or social desirability biases. Physical bullying in school is relatively more common and familiar to teachers and parents. Non-physical bullying (e.g., social exclusion) is less visible and more easily neglected. In addition, with the rapid development of Internet and electronic technology, children and adolescents are more likely to be exposed to bullying in an electronic context (e.g., e-mail, blogs, instant messages, text messages). Hence, longitudinal studies are needed to explore the associations of non-physical and physical bullying with suicidal ideation and attempt.

## Conclusions

Despite its limitations, our study clearly described the associations of being threatened or injured with a weapon in school and suicidal ideation and attempt among middle and high school students in Zhejiang China. We found that being threatened or injured with a weapon in school was common, and was strongly associated with both suicidal ideation and attempt. Preventive measures for physical bullying among middle and high school students need to be reinforced in Zhejiang China, and efforts to prevent adolescent suicide may need to address school bullying. Suicide prevention should be an important component of psychological intervention for threatened or injured students.

## Abbreviations

CDC: Disease Control and Prevention
CI: Confidence Intervals
NCDs: Non-Communicable Diseases
SD: Standard Deviation
USA: United States of America
WHO: World Health Organization
YRBS: Youth Risk Behaviour Survey
